# Dietary Carbohydrates, Genetic Susceptibility, and Gout Risk: A Prospective Cohort Study in the UK

**DOI:** 10.3390/nu16172883

**Published:** 2024-08-28

**Authors:** Baojie Hua, Ziwei Dong, Yudan Yang, Wei Liu, Shuhui Chen, Ying Chen, Xiaohui Sun, Ding Ye, Jiayu Li, Yingying Mao

**Affiliations:** Department of Epidemiology, School of Public Health, Zhejiang Chinese Medical University, Hangzhou 310053, China

**Keywords:** dietary carbohydrates, gout, genetic risk, biomarkers, mediation analysis, cohort study

## Abstract

This study aimed to investigate the associations between carbohydrate intake and gout risk, along with interactions between genetic susceptibility and carbohydrates, and the mediating roles of biomarkers. We included 187,387 participants who were free of gout at baseline and completed at least one dietary assessment in the UK Biobank. Cox proportional hazard models were used to estimate the associations between carbohydrate intake and gout risk. Over a median follow-up of 11.69 years, 2548 incident cases of gout were recorded. Total carbohydrate intake was associated with a reduced gout risk (Q4 vs. Q1: HR 0.67, 95% CI 0.60–0.74), as were total sugars (0.89, 0.80–0.99), non-free sugars (0.70, 0.63–0.78), total starch (0.70, 0.63–0.78), refined grain starch (0.85, 0.76–0.95), wholegrain starch (0.73, 0.65–0.82), and fiber (0.72, 0.64–0.80), whereas free sugars (1.15, 1.04–1.28) were associated with an increased risk. Significant additive interactions were found between total carbohydrates and genetic risk, as well as between total starch and genetic risk. Serum urate was identified as a significant mediator in all associations between carbohydrate intake (total, different types, and sources) and gout risk. In conclusion, total carbohydrate and different types and sources of carbohydrate (excluding free sugars) intake were associated with a reduced risk of gout.

## 1. Introduction

Gout is the most common form of inflammatory arthritis which affects between 1% and 4% of people in Europe, and manifests increased incidence and prevalence across the globe [1]. It is characterized by the persistent increase of serum uric acid, the saturation of urate in body tissues, and the accumulation of monosodium urate crystals in and around the joints [1], and finally triggers recurrent episodes of arthritis and progressive joint injury [2]. Importantly, gout has been linked to many chronic conditions such as cardiovascular disease [3], venous thromboembolism [4], and metabolic syndrome [5], which exacerbates the public health burden.

Diet plays an important role in the pathogenesis of gout [6]. Carbohydrates, as crucial nutrients in our diet, provide energy to our bodies. However, there has been an ongoing debate about the link between carbohydrate intake and health outcomes [7]. For instance, a low-carbohydrate diet, which involves substituting carbohydrates with higher protein or fat intake, gained popularity as a weight-loss strategy [8,9]. Nevertheless, a meta-analysis of cohort studies including 432,179 participants found a U-shaped association between carbohydrate consumption and all-cause mortality [10]. Though research has shown that low-carbohydrate weight-loss diets can reduce serum urate levels [11], it is still unclear whether carbohydrate intake has an impact on the risk of gout.

Recent studies have indicated that, apart from the quantity of carbohydrates, the specific types of carbohydrate consumed had a more significant impact on health outcomes. Carbohydrates can be classified according to their structure and functions into three types: sugars, starch, and fiber. Sugars are simple carbohydrates and are the fastest form of energy. They include free sugars and non-free sugars. An example is fructose, which is found in fruit (such as apples and pears), honey, and some vegetables. On the other hand, starch is a complex carbohydrate made up of long chains of glucose. It serves as a form of energy storage in plants. One food high in starch is potatoes. Fiber is a type of carbohydrate that cannot be digested by the human body. It is crucial for digestive health and has other health benefits. Some examples are cellulose, hemicellulose, and pectin [12,13]. The relationship between fructose and the risk of gout has been well established. Previous meta-analysis of prospective cohort studies with more than 100,000 participants found that fructose intake [14], fruit juice intake, and sugar-sweetened beverage intake [15] increased the likelihood of gout. The latest American College of Rheumatology Guideline for the management of gout also conditionally recommended limiting the consumption of high-fructose corn syrup [16]. However, there has been limited epidemiological evidence regarding the effects of dietary fiber and starch on the risk of gout. Only one case-control study showed that dietary fiber might have a protective effect against gout risk [17].

The interplays between diet and genetic factors in the etiology of gout have been under-investigated. A prospective study suggested that there was a significant additive interaction effect between unhealthy dietary pattern and genetic predisposition to gout risk [18]. However, evidence regarding the interactions between dietary carbohydrates and genetic susceptibility in relation to gout risk was limited.

The hypotheses of this study are as follows: total carbohydrate intake may influence the risk of gout, with different types and sources of carbohydrate potentially having varying effects, possibly mediated by several biomarkers. Additionally, given the significant role of genetic factors in gout, there may be interactive effects between genetic susceptibility and dietary carbohydrates on gout risk. Therefore, our study aimed to investigate the relationships between total carbohydrate intake and the risk of gout, as well as the effects of carbohydrates from different types and sources, leveraging data from a large-scale cohort study. We also aimed to examine the combined effects of dietary carbohydrates and genetic susceptibility on gout risk, along with potential interactions between these factors. Additionally, we explored whether blood and urine biomarkers might play mediating roles in the associations between dietary carbohydrates and gout risk.

## 2. Methods

### 2.1. Study Participants

The UK Biobank (UKB) is a large-scale prospective study that enrolled over 500,000 individuals aged 40–69 years between 2006 and 2010. This study collected comprehensive data on socio-demographic factors, health behaviors, and medical history through a combination of touch-screen questionnaires and interviews at the Assessment Centre [19]. Additionally, body size measures (such as height and weight) were collected using standard measuring devices, and detailed measurement protocols can be found at https://biobank.ndph.ox.ac.uk/ukb/refer.cgi?id=146620 (accessed on 19 August 2024). Biological samples (such as blood and urine) were collected by trained staff. Detailed information on the collection, processing, and storage of biological samples can be found at https://biobank.ndph.ox.ac.uk/ukb/refer.cgi?id=100226 (accessed on 19 August 2024). Both physical measures and biological samples were collected from all participants at the UKB Assessment Centre between 2006 and 2010.

Ethical approval for the UKB study was granted by the North West Multi-Center Research Ethics Committee in the UK and the Community Health Index Advisory Group in Scotland. The study was conducted in compliance with the general ethical approval provided by the National Research Ethics Service. Before participating in the study, all individuals provided written informed consent.

We performed a prospective cohort study based on UKB. As shown in Figure S1, a total of 502,415 participants were obtained, and after excluding withdrawn individuals (N = 48), participants with prevalent gout (N = 1167), without any valid 24-h dietary assessment data (N = 290,658), or having missing covariate information (N = 23,155), a total of 187,387 participants were finally included in our main analysis. In the subsequent genetic analyses, we excluded 142 participants with sex mismatch, and 55,752 participants with genetic kinship, resulting in 131,493 participants available to study the interplays between dietary carbohydrates and genetic susceptibility. Additionally, for the mediation analyses, we excluded participants with missing biomarker data. Due to variations in the amount of missing data across different biomarkers, the final sample size for the mediation analyses ranged from 141,978 to 182,311.

### 2.2. Measurement of Dietary Carbohydrates Intake

Between 2009 and 2012, a web-based 24-h dietary instrument called the Oxford WebQ was utilized to collect information on the consumption of 206 types of food and 32 types of beverages. Data were collected either at the Assessment Centre (instance 0) or online (instance 1–4) [20]. Unlike traditional 24-h dietary recalls that rely on respondents recalling and reporting consumed foods, the Oxford WebQ requires individuals to specify the type of food and the quantity from a pre-set list of 21 food groups using standard serving categories or portions. Comparisons between the dietary assessment data collected by an interviewer and the data recorded by the Oxford WebQ showed moderate-to-strong correlations for most nutrients [21]. Therefore, the Oxford WebQ can be described as a combination of a 24-h dietary recall and a food frequency questionnaire [22]. To estimate the intake of food nutrients, the Oxford WebQ automatically calculated the data by multiplying the number of portions consumed by the predetermined quantity of each food portion size and its corresponding nutrient composition. The nutrient composition information used was derived from the UK Nutrient Databank food composition tables [23].

For individuals with only one dietary measurement, the intake was considered based on that single measurement. For individuals with two or more dietary measurements, we averaged the intake by summing all recorded intakes and dividing by the total number of assessments. Based on previous studies [24,25], the types of carbohydrate analyzed included total sugars, starch, and fiber. Among them, total sugars were further categorized into free sugars and non-free sugars. The definition of free sugars included all monosaccharides and disaccharides that were added to food by manufacturers, chefs, or consumers, as well as sugars naturally present in honey, syrups, and unsweetened fruit juices. Non-free sugars refer to sugars that naturally exist in foods, with fruit, vegetables, and dairy products being the main dietary sources [26]. The sources of starch were calculated separately for refined grain and wholegrain. The approximate calculation of the starch content in wholegrain and refined grain foods was based on the consumption of wholegrain and refined grains, considering that starch serves as the principal constituent of wheat grains [27]. More specific information on the food items used to calculate the intake of different types and sources of carbohydrate is shown in Table S1. Energy adjustment for dietary carbohydrates was conducted using the residual method [28].

### 2.3. Ascertainment of Gout

The primary outcome of this study was gout, which was ascertained using codes from the 9th and 10th revisions of the International Classification of Diseases (ICD). Specifically, the ICD-9 code was 2749, and the ICD-10 code was M10. Incident gout cases were identified based on the first diagnosis recorded in the hospital inpatient database during the follow-up period.

### 2.4. Genetic Risk Score Calculation

The genotyping of the study participants utilized two arrays: the UK BiLEVE array and the UKB Axiom array. The BiLEVE array was initially tested on around 50,000 participants, while the UKB Axiom array was used for the remaining approximately 450,000 participants. Further information regarding the genotyping process, imputation, and quality control measures can be found in the previous study [29]. In the present study, the genetic risk score (GRS) for gout was constructed using 33 genetic susceptibility loci achieving genome-wide significance (*p* < 5 × 10^−8^) [30] (Table S2). Every single nucleotide polymorphism (SNP) was recoded as zero, one, or two, according to the number of effect alleles to construct the GRS. The GRS was derived as *β_1_* × SNP_1_ + *β_2_* × SNP_2_ + … + *β_k_* × SNP_k_ + … + *β_n_* × SNP_n_, of which n was the number of SNPs, and *β_k_* was the Ln (odds ratio) for the association between gout and SNP_k_.

### 2.5. Measurements of Covariates and Biomarkers

Information on sociodemographic and lifestyle factors was collected at baseline using self-reported touch-screen questionnaires. Body mass index (BMI) was calculated as body weight (kilogram, kg) divided by the square of height (meters, m). Physical activity was defined as participants having regular physical activity in the form of more than 150 min of moderate activity per week, or more than 75 min of vigorous activity per week, or at least 5 days of moderate physical activity per week, or one vigorous activity per week [31].

UKB analyzed a diverse array of biochemical markers in approximately 480,000 participant samples from the recruitment visit. The selection of assays was conducted by both internal and external experts, with 34 biomarkers ultimately tested by the UKB. For more comprehensive information on the biomarker measurements and quality control procedures carried out by UKB, additional details can be accessed online (https://biobank.ndph.ox.ac.uk/ukb/label.cgi?id=17518, accessed on 9 January 2024; https://biobank.ndph.ox.ac.uk/ukb/label.cgi?id=100083, accessed on 9 January 2024). For mediation analyses, we excluded those biomarkers with missing values exceeding 70%, resulting in a final set of 30 biomarkers, including 28 blood biomarkers and 2 urine biomarkers (Table S3).

### 2.6. Statistical Analysis

All statistical analyses were conducted using R software version 4.2.3 and Statistics Analysis System (SAS) software version 9.4. The Cox proportional hazards model was utilized to assess the associations between energy-adjusted dietary carbohydrates and gout risk. Model 1 was adjusted for age (continuous) and sex (male or female). Model 2 was further adjusted for ethnicity (white or other), household income (<£18,000, £18,000−£30,999, £31,000−£51,999, £52,000−£100,000, or >£100,000), education (high, medium, or other), BMI (continuous, kg/m^2^), smoking status (never, previous, or current), alcohol drinking status (never, previous, or current), and physical activity (yes or no). Energy-adjusted dietary carbohydrate intake was categorized into quartiles, with the lowest quartile as the reference group. Linear trends were assessed by treating the quartiles as continuous variables. Hazard ratios (HRs) and 95% confidence intervals (95% CIs) for per interquartile range (IQR) increase of dietary carbohydrate intake were also estimated.

To further evaluate the robustness of our findings, we conducted a series of sensitivity analyses, which included: (1) excluding participants diagnosed with gout within the first two years of follow-up; (2) excluding participants diagnosed with gout before the last 24-h dietary assessment; (3) excluding participants with only one 24-h dietary assessment; (4) restricting the analyses to participants with fairly typical diets during the 24-h dietary assessments; (5) excluding participants with extreme energy intake (Males: <3347 kJ/d or >17,573 kJ/d; Females: <2092 kJ/d or >14,644 kJ/d) [25]; (6) further adjusting for prevalent diabetes, prevalent hypertension, and prevalent cardiovascular disease; (7) further adjusting for the use of diuretics, anti-hypertensive drugs, anti-hyperlipidemic drugs, and anti-diabetic drugs; (8) rerunning the analyses after multiple imputations for missing covariates.

A restricted cubic spline model was employed to investigate the potential dose–response relationships between energy-adjusted dietary carbohydrates and the risk of gout. The number of knots was chosen based on Akaike’s information criterion. Stratified analyses were conducted across various factors including age (<60 or ≥60 years), sex (male or female), and BMI (<25 kg/m^2^ or ≥25 kg/m^2^).

Furthermore, we assessed the joint effects of energy-adjusted dietary carbohydrates and genetic susceptibility on the risk of gout. We categorized GRS into three groups: low, intermediate, and high genetic risk. Concerning free sugars, the reference group consisted of individuals with a low GRS and a low intake of free sugars. In the case of other carbohydrates, the reference group was individuals with a high GRS and a low level of carbohydrate intake. We evaluated both additive and multiplicative interaction, of which additive interaction was assessed using the relative excess risk due to interaction (RERI) and the attributable proportion due to the interaction (AP), and multiplicative interaction was examined by including cross-product terms in the Cox proportional hazards model.

We screened biomarkers for mediation analysis in the following steps: firstly, we utilized quantile regression on the 25th, 50th, and 75th quantiles to examine the relationships between dietary carbohydrates and biomarkers. Subsequently, the Cox proportional hazards model was employed to evaluate the associations between biomarkers and the risk of gout. Finally, we selected biomarkers that exhibited significant associations on both aforementioned steps for further mediation analyses, which were conducted using the SAS macro program “%mediate” [32].

## 3. Results

Baseline characteristics of study participants are shown across the lowest and the highest quartiles of dietary total carbohydrate intake. As listed in Table 1, compared to the participants with the lowest total carbohydrate intake, those with the highest intake were more likely to have a lower household income, be non-alcohol drinkers, have higher energy intake, and engage in physical activity. There were strong correlations between total carbohydrates and total sugars (r^2^ = 0.81, *p* < 0.001), and between total carbohydrates and total starch (r^2^ = 0.81, *p* < 0.001) (Table S4).

During a median follow-up period of 11.69 years, 2548 incident gout cases were identified. Associations of energy-adjusted dietary carbohydrate intake with the risk of gout are shown in Table 2. The fully adjusted HRs with 95% CIs for gout risk among participants in the highest quartiles (Q4) [compared to the lowest quartiles (Q1)] of energy-adjusted dietary carbohydrate intake were as follows: 0.67 (0.60–0.74) for total carbohydrates (*P_trend_* = 3.073 × 10^−15^), 0.89 (0.80–0.99) for total sugars (*P_trend_* = 0.018), 1.15 (1.04–1.28) for free sugars (*P_trend_* = 0.014), 0.70 (0.63–0.78) for non-free sugars (*P_trend_* = 2.100 × 10^−11^), 0.70 (0.63–0.78) for total starch (*P_trend_* = 7.254 × 10^−12^), 0.85 (0.76–0.95) for refined grain starch (*P_trend_* = 0.002), 0.73 (0.65–0.82) for wholegrain starch (*P_trend_* = 1.536 × 10^−9^), and 0.72 (0.64–0.80) for fiber (*P_trend_* = 2.638 × 10^−9^). These findings remained robust in sensitivity analyses (Tables S5–S12), except for the non-significant association between free sugars and gout in sensitivity analyses conducted for participants with at least two 24-h dietary assessments (Table S7).

**Figure 1:**
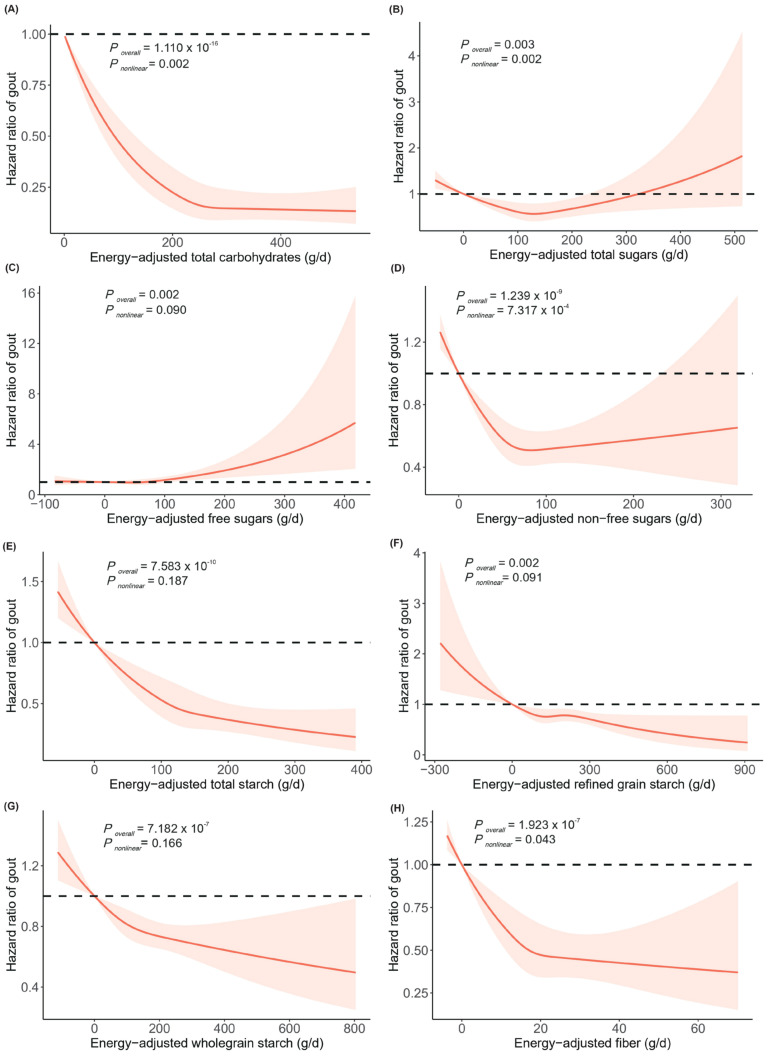
Dose–response relationships between energy-adjusted total carbohydrates (**A**), total sugars (**B**), free sugars (**C**), non-free sugars (**D**), total starch (**E**), refined grain starch (**F**), wholegrain starch (**G**), and fiber (**H**) and the risk of gout. The lines represent adjusted hazard ratios (solid lines) and 95% confidence intervals (dashed lines) based on the restricted cubic spline models for energy-adjusted carbohydrate intake. The adjusted covariates included age, sex, ethnicity, household income, education, BMI, smoking status, alcohol drinking status, and physical activity. Energy-adjusted carbohydrates are presented on the *x*–axis, excluding the values outside the 2.5th percentiles and 97.5th percentiles, and hazard ratios of gout are presented on the *y*–axis. Abbreviations: BMI, body mass index.

Figure 1 illustrates the potential dose–response relationships between dietary carbohydrate intake and the risk of gout. Non-linear associations were observed for the intake of total carbohydrates, total sugars, non-free sugars, and fiber with gout risk (all *P_overall_* < 0.050, all *P_non-linear_* < 0.050). We also investigated whether the associations between dietary carbohydrates and gout risk were consistent across various subgroups defined by age, sex, and BMI (Tables S13–S15). We found significant evidence suggesting effect modification by BMI of the associations of total carbohydrates (*P_heterogeneity_* = 0.003), non-free sugar intake (*P_heterogeneity_* = 0.018), and total starch (*P_heterogeneity_* = 0.003) with gout risk (Table S15).

**Figure 2:**
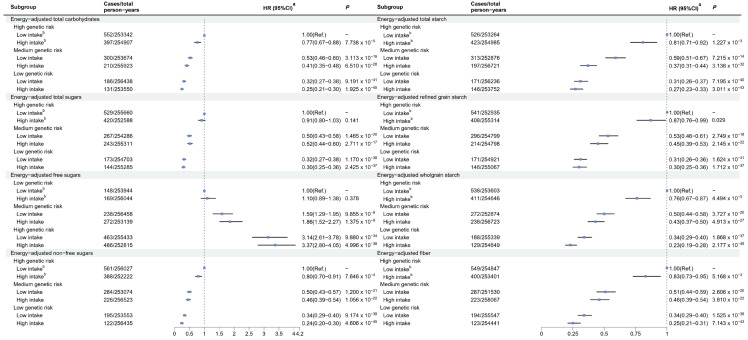
Joint effects of dietary carbohydrates and genetic risk score on the risk of gout. ^a^ Adjusted for age, sex, ethnicity, household income, education, BMI, smoking status, alcohol drinking status, and physical activity. ^b^ Dietary energy-adjusted carbohydrate intakes were divided into high and low levels based on the median: total carbohydrates (≥256.64 vs. <256.64 g/d), total sugars (≥122.90 vs. <122.90 g/d), free sugars (≥57.77 vs. <57.77 g/d), non-free sugars (≥60.55 vs. <60.55 g/d), total starch (≥129.13 vs. <129.13 g/d), refined grain starch (≥153.38 vs. <153.38 g/d), wholegrain starch (≥96.24 vs. <96.24 g/d), and fiber (≥17.43 vs. <17.43 g/d). Abbreviations: BMI, body mass index; Ref., Reference.

The GRS of gout was positively associated with the risk of gout (Table S16). As shown in Figure 2, compared to participants with high genetic risk and a low intake of dietary carbohydrates, those with low genetic risk and high intake of total carbohydrates (HR 0.25, 95% CI 0.21–0.30), total sugars (HR 0.30, 95% CI 0.25–0.36), non-free sugars (HR 0.24, 95% CI 0.20–0.30), total starch (HR 0.27, 95% CI 0.23–0.33), refined grain starch (HR 0.30, 95% CI 0.25–0.36), wholegrain starch (HR 0.23, 95% CI 0.19–0.28), and fiber (HR 0.25, 95% CI 0.21–0.31) had the lowest risk of gout. Conversely, compared to participants with low genetic risk and low intake of dietary free sugars, those with high genetic risk and high intake of free sugars (HR 3.37, 95% CI 2.80–4.05) had the highest risk of gout. We did not observe any multiplicative interactions between GRS and dietary carbohydrate intake (Table S17). However, there were negative additive interactions between total carbohydrate intake and high genetic risk (RERI = −0.49, 95% CI −0.92 to −0.05; AP = −0.20, 95% CI −0.39 to −0.02), as well as between total starch intake and both moderate and high genetic risk (RERI = −0.57, 95% CI −0.96 to −0.19; AP = −0.48, 95% CI −0.80 to −0.16; RERI = −0.49, 95% CI −0.94 to −0.03; AP = −0.19, 95% CI −0.36 to −0.01, respectively) (Table S17).

The results of the associations between dietary carbohydrates and biomarkers, as well as biomarkers and gout risk, are shown in Tables S18 and S19, respectively, and 22 biomarkers were ultimately included in the mediation analyses. Figure S2 and Table S20 display the potential mediating effects of blood and urine biomarkers on the associations between dietary carbohydrates and gout risk. Among the 22 biomarkers, 18 (81.8%) showed statistically significant mediation effects (*p* < 0.050), with 11 (50.0%) still demonstrating significant mediating effects after Bonferroni correction (*p* < 2.273 × 10^−3^). Serum urate (SUA) was identified as a mediator for total carbohydrates and different types and sources of carbohydrate intake, with the mediating proportion ranging from 22.8% to 83.1%. Among different types of carbohydrate, wholegrain starch intake had the highest number of mediators (N = 16). The range of mediating proportion for this relationship was between 6.1% and 53.8%.

## 4. Discussion

In this large prospective cohort study, the intake of total carbohydrates, total sugars, non-free sugars, starch, and fiber were inversely associated with the risk of gout, while intake of free sugars was linked to an elevated risk of gout. Participants with low genetic risk and higher intake of total carbohydrates, total sugars, non-free sugars, starch, and fiber had the lowest risk of gout, while the highest gout risk was observed among participants with high free sugar intake and high genetic risk. Negative additive interactions were observed between total carbohydrate intake and high genetic risk, as well as between total starch intake and both moderate and high genetic risk. In addition, 11 blood and urine biomarkers were found to mediate the associations between dietary carbohydrates and gout risk, among which SUA played the most significant mediating role in these relationships.

In this study, we observed that a higher intake of total carbohydrates was associated with a decreased risk of gout. Despite the limited epidemiological evidence on the association between total carbohydrates and gout, previous studies identified a relationship between total carbohydrate intake and uric acid, which is the primary biomarker for gout. For example, a cross-sectional study revealed a negative association between total carbohydrate intake and serum uric acid levels [33]. Moreover, a clinical trial revealed that participants who consumed low glycemic index carbohydrates had lower plasma uric acid levels [34]. On the other hand, the total energy intake of an individual is relatively fixed, so when carbohydrate intake increases, the intake of protein and fats tends to decrease accordingly. Previous studies have found that low carbohydrate, high protein, and high fat intakes were associated with an increased risk of gout [35,36], suggesting a protective effect of high carbohydrate intake. In the current study, compared to the low carbohydrate intake group (Q1), the energy from protein and fat (% of total energy intake) in the high carbohydrate intake group (Q4) decreased (protein Q4 vs. Q1: 14.49% vs. 18.61%; fat Q4 vs. Q1: 31.68% vs. 34.05%), which might help explain the protective effect of carbohydrates against gout.

Interestingly, our study suggested that a high intake of non-free sugars was associated with a reduced risk of gout, while free sugars had the opposite effect. Currently, there is a lack of research on non-free sugars and gout. Previous studies on food sources rich in non-free sugars (fruit, vegetables, and dairy products [26]) have suggested a protective effect on gout risk, which partially supports our findings. For example, a prospective study of 28,990 males in the United States found that males who consumed an average of more than two pieces of fruit per day had a 50% lower risk of gout compared to those who consumed less than half a piece of fruit per day [37]. Another prospective study involving 47,150 American men also found that the incidence of gout reduced with an increased intake of dairy products [38]. Regarding free sugars, our findings align with previous observational studies that have shown a significant association between free sugar consumption and an increased risk of gout [39]. Additionally, several meta-analyses have found a significant positive association between the consumption of sugar-sweetened beverages (rich in free sugars) and gout risk [15,40,41].

Our findings showed a negative association between dietary fiber and the risk of gout. Similarly, a previous observational study conducted among 66,427 Chinese adults identified an association between a high-fiber diet and a reduced prevalence of hyperuricemia, which is a precursor to the development of gout [42]. Mechanistically, dietary fiber has been found to reduce SUA concentration by slowing and inhibiting the organ’s digestion and/or absorption of dietary purines or adenosine [43]. In a gout-like mouse model, a high-fiber diet was found to modulate the inflammatory response in gout characterized by neutrophil infiltration, neutrophil-dependent tissue damage, and joint dysfunction caused by monosodium uric acid crystals. This modulation occurs through an increase in short-chain fatty acids, promoting caspase-dependent neutrophil apoptosis, efferocytosis, and the subsequent resolution of inflammation [44].

We found that the consumption of both wholegrain starch and refined grain starch were associated with a lower risk of gout. A prospective cohort study based on the Health Professionals Follow-up Study involving 44,444 males found the Dietary Approaches to Stop Hypertension diet characterized by rich wholegrains was associated with a lower risk of gout [45]. In addition, several studies have found a significant association between the consumption of wholegrains and a reduced risk of cardiometabolic diseases such as CVD [46,47] and diabetes [48,49], which were closely associated with gout [5]. Refined grain intake was once assumed to be associated with adverse health outcomes, however, in recent years the relationship between refined grain consumption and health has become a topic of debate. For example, a cross-sectional study in Chinese adults found that a 10-g increase in refined grain consumption was associated with 0.9% reduced risk of hyperuricemia [50], which is consistent with our findings.

In the current study, SUA acted as the most significant mediator in all the associations between dietary carbohydrates and gout. Since hyperuricemia is a recognized precursor to gout, these findings further strengthen the relationship between dietary carbohydrate intake and gout risk. In addition, cystatin C (CYS) and gamma-glutamyl transferase (GGT) are two other important mediating factors. We observed a positive association between total carbohydrate intake and CYS levels. Similarly, a study of 307 obese adults in the United States showed that a low-carbohydrate, high-protein diet was linked to reduced CYS levels [51]. Additionally, a case-control study of 326 gout patients and 210 healthy controls found that gout patients had higher CYS levels [52]. The aforementioned evidence supports the mediating role of CYS in the relationship between carbohydrates and gout. Regarding GGT, a cohort study with 3146 participants found negative associations between dairy products, vegetables, fruit, refined grains, and wholegrains and GGT levels, aligning with our findings [53]. Elevated serum GGT levels may contribute to hyperuricemia [54]. Thus, the evidence suggests that GGT may act as a mediator in the relationship between carbohydrates and gout. However, further research is needed to fully understand how these mediators influence the relationship between dietary carbohydrates and gout.

Our research has important public health implications for gout prevention. We found that different types and sources of carbohydrate provide a protective effect against gout, while free sugars have the opposite effect. Therefore, individuals can reduce their risk of gout by enhancing their consumption of foods abundant in beneficial carbohydrates while restricting those rich in free sugars. Moreover, it is crucial to implement stratified management strategies that account for individuals’ genetic susceptibilities. For those at high genetic risk of gout, increasing the intake of protective carbohydrates and reducing the intake of harmful carbohydrates is essential. In addition to focusing on carbohydrate consumption, gout prevention should adopt a multifaceted approach. Previous studies have suggest that increasing the intake of plant-based foods [55]—including vegetables, fruit, and legumes—along with adhering to healthy lifestyle factors (such as moderate alcohol consumption, not smoking, regular physical activity, and a balanced diet) [56] can further reduce the risk of gout. This comprehensive strategy can help alleviate the medical and economic burden of gout on society.

This study had several strengths. Firstly, it was a population-based prospective cohort study design with a large sample size. Secondly, it examined the joint and interactive effects of dietary carbohydrate intake and genetic risk on gout risk. Lastly, it considered the mediating roles of multiple biomarkers in the relationship between dietary carbohydrates and gout risk. However, this study also had some limitations. First, dietary intake data and most covariate information were self-reported by participants, which may have introduced information bias. Secondly, because dietary measurements in the UKB were conducted over a longer period, we were unable to compare the effects of dietary carbohydrate intake measured over a short period versus a longer period on gout risk. Thirdly, this study was observational in nature, and causal relationships need further confirmation. Finally, the participants of this study were from the UKB, limiting the generalization of the findings to other populations, so further research support is needed.

## 5. Conclusions

In this study, the risk of gout was found to have inverse associations with the intake of dietary total carbohydrates, total sugars, non-free sugars, starch, and fiber, while the intake of free sugars was positively associated with gout risk. Our findings offer valuable insights to help individuals reduce their risk of gout through dietary carbohydrate adjustments, ultimately alleviating the associated public health burden.

## Figures and Tables

**Table 1:** Baseline characteristics across the lowest and highest quartiles of energy-adjusted total carbohydrate intake.

Characteristics	Energy-Adjusted Total Carbohydrate Intake (g/d) ^a^			
	Q1 (<230.50)	Q2 (≥230.50 & <256.73)	Q3 (≥256.73 & <281.42)	Q4 (≥281.42)
N	46,847	46,847	46,846	46,847
Age (years), median (IQR)	57.0 (13.0)	57.0 (12.0)	57.0 (12.0)	57.0 (14.0)
Sex, *n* (%)				
Female	23,204 (49.5)	26,754 (57.1)	26,832 (57.3)	23,804 (50.8)
Male	23,643 (50.5)	20,093 (42.9)	20,014 (42.7)	23,043 (49.2)
Ethnicity, *n* (%)				
White	45,349 (96.8)	45,183 (96.4)	45,023 (96.1)	43,980 (93.9)
Other	1498 (3.2)	1664 (3.6)	1823 (3.9)	2867 (6.1)
Education, *n* (%)				
High (college or university degree, professional qualifications)	3064 (6.5)	3350 (7.2)	3611 (7.7)	4273 (9.1)
Medium (A level/AS level, O level/GCSE, CSE, NVQ or HND or HNC)	19,731 (42.1)	20,171 (43.1)	20,182 (43.1)	20,525 (43.8)
Other	24,052 (51.3)	23,326 (49.8)	23,053 (49.2)	22,049 (47.1)
BMI (kg/m^2^), median (IQR)	26.7 (5.6)	26.2 (5.5)	26.0 (5.5)	26.1 (5.5)
Household income, *n* (%)				
<£18,000	5641 (12.0)	6688 (14.3)	7653 (16.3)	9057 (19.3)
£18,000–£30,999	9926 (21.2)	11,272 (24.1)	11,881 (25.4)	12,493 (26.7)
£31,000–£51,999	13,422 (28.7)	13,458 (28.7)	13,465 (28.7)	13,125 (28.0)
£52,000–£100,000	12,920 (27.6)	11,947 (25.5)	10,934 (23.3)	9965 (21.3)
>£100,000	4938 (10.5)	3482 (7.4)	2913 (6.2)	2207 (4.7)
Physical activity, *n* (%)				
No	12,955 (27.7)	12,500 (26.7)	12,158 (26.0)	11,394 (24.3)
Yes	33,892 (72.3)	34,347 (73.3)	34,688 (74.0)	35,453 (75.7)
Smoking status, *n* (%)				
Never	22,087 (47.1)	25,986 (55.5)	28,262 (60.3)	29,179 (62.3)
Previous	19,525 (41.7)	17,173 (36.7)	15,576 (33.2)	14,670 (31.3)
Current	5235 (11.2)	3688 (7.9)	3008 (6.4)	2998 (6.4)
Alcohol drinking status, *n* (%)				
Never	444 (0.9)	938 (2.0)	1598 (3.4)	2663 (5.7)
Previous	637 (1.4)	1077 (2.3)	1521 (3.2)	2365 (5.0)
Current	45,766 (97.7)	44,832 (95.7)	43,727 (93.3)	41,819 (89.3)
Energy (kJ/d), median (IQR)	8777.2 (3259.0)	8105.9 (2818.8)	8098.0 (2726.8)	8782.9 (3028.4)
Energy-adjusted total sugars (g/d), median (IQR)	96.4 (34.6)	116.7 (31.5)	131.2 (33.9)	154.3 (46.3)
Energy-adjusted free sugars (g/d), median (IQR)	47.2 (28.6)	55.6 (27.6)	61.2 (30.37)	70.0 (42.2)
Energy-adjusted non-free sugars (g/d), median (IQR)	45.1 (28.0)	57.5 (28.7)	66.2 (31.8)	78.2 (41.8)
Energy-adjusted total starch (g/d), median (IQR)	107.2 (36.1)	127.3 (31.3)	137.0 (33.3)	147.3 (43.3)
Energy-adjusted refined grain starch (g/d), median (IQR)	120.2 (108.0)	153.1 (111.1)	165.7 (121.7)	181.8 (158.0)
Energy-adjusted wholegrain starch (g/d), median (IQR)	71.2 (100.8)	94.3 (107.7)	107.6 (113.9)	118.9 (138.6)
Energy-adjusted fiber (g/d), median (IQR)	14.6 (5.8)	16.9 (5.5)	18.4 (6.0)	20.2 (7.4)

^a^ Energy adjustment for total carbohydrates was conducted using the residual method. Abbreviations: A level, Advanced level; AS level, Advanced Subsidiary level; BMI, body mass index; GCSE, General Certificate of Secondary Education; CSE, Certificate of Secondary Education; HNC, Higher National Certificate; HND, Higher National Diploma; IQR, interquartile range; O level, Ordinary level; NVQ, National Vocational Qualification.

**Table 2:** Hazard ratios (95% confidence intervals) for the associations between dietary carbohydrates and the risk of gout.

	Quartiles of Energy-Adjusted Dietary Carbohydrates (g/d) ^a^				*P_trend_*	Per IQR Increase
	Q1	Q2	Q3	Q4		
Energy-adjusted total carbohydrates (g/d)	<230.50	≥230.50 & <256.73	≥256.73 & <281.42	≥281.42		
Cases/total person-years	899/543,581	606/545,461	515/546,611	528/543,926		2548/2,179,579
Model 1	1.00 (Ref.)	0.74 (0.67–0.82)	0.62 (0.56–0.69)	0.59 (0.53–0.66)	6.068 × 10^−25^	0.78 (0.75–0.82)
Model 2	1.00 (Ref.)	0.79 (0.71–0.88)	0.69 (0.62–0.77)	0.67 (0.60–0.74)	3.073 × 10^−15^	0.83 (0.79–0.87)
Energy-adjusted total sugars (g/d)	<100.97	≥100.97 & <122.91	≥122.91 & <146.72	≥146.72		
Cases/total person-years	809/544,383	596/546,234	541/545,863	602/543,099		2548/2,179,579
Model 1	1.00 (Ref.)	0.77 (0.69–0.86)	0.71 (0.64–0.80)	0.77 (0.69–0.86)	2.525 × 10^−7^	0.88 (0.84–0.92)
Model 2	1.00 (Ref.)	0.84 (0.75–0.93)	0.82 (0.74–0.92)	0.89 (0.80–0.99)	0.018	0.94 (0.90–0.98)
Energy-adjusted free sugars (g/d)	<42.44	≥42.44 & <57.73	≥57.73 & <75.61	≥75.61		
Cases/total person-years	649/545,507	579/546,605	572/546,010	748/541,457		2548/2,179,579
Model 1 ^b^	1.00 (Ref.)	0.95 (0.85–1.06)	0.90 (0.80–1.00)	1.07 (0.96–1.18)	0.355	1.04 (1.00–1.08)
Model 2 ^c^	1.00 (Ref.)	1.01 (0.90–1.13)	0.99 (0.88–1.11)	1.15 (1.04–1.28)	0.014	1.06 (1.02–1.10)
Energy-adjusted non-free sugars (g/d)	<43.88	≥43.88 & <60.70	≥60.70 & <80.04	≥80.04		
Cases/total person-years	889/542,125	625/545,734	538/546,193	496/545,527		2548/2,179,579
Model 1	1.00 (Ref.)	0.72 (0.65–0.80)	0.64 (0.58–0.71)	0.63 (0.56–0.70)	4.423 × 10^−19^	0.78 (0.74–0.83)
Model 2	1.00 (Ref.)	0.78 (0.71–0.87)	0.72 (0.64–0.80)	0.70 (0.63–0.78)	2.100 × 10^−11^	0.83 (0.79–0.88)
Energy-adjusted total starch (g/d)	<109.29	≥109.29 & <129.26	≥129.26 & <149.44	≥149.44		
Cases/total person-years	821/541,939	608/545,723	542/546,855	577/545,062		2548/2,179,579
Model 1	1.00 (Ref.)	0.77 (0.69–0.85)	0.68 (0.61–0.76)	0.68 (0.61–0.76)	5.703 × 10^−14^	0.82 (0.79–0.86)
Model 2	1.00 (Ref.)	0.81 (0.73–0.90)	0.72 (0.65–0.80)	0.70 (0.63–0.78)	7.254 × 10^−12^	0.84 (0.81–0.88)
Energy-adjusted refined grain starch (g/d)	<96.21	≥96.21 & <153.17	≥153.17 & <222.30	≥222.30		
Cases/total person-years	809/542,874	623/545,155	576/546,047	540/545,504		2548/2,179,579
Model 1	1.00 (Ref.)	0.91 (0.82–1.01)	0.88 (0.79–0.98)	0.87 (0.78–0.97)	0.006	0.92 (0.88–0.97)
Model 2	1.00 (Ref.)	0.91 (0.82–1.02)	0.88 (0.79–0.98)	0.85 (0.76–0.95)	0.002	0.92 (0.88–0.96)
Energy-adjusted wholegrain starch (g/d)	<45.99	≥45.99 & <96.03	≥96.03 & <162.67	≥162.67		
Cases/total person-years	774/541,364	643/545,601	583/546,386	548/546,228		2548/2,179,579
Model 1	1.00 (Ref.)	0.85 (0.77–0.94)	0.69 (0.62–0.77)	0.59 (0.53–0.66)	3.553 × 10^−24^	0.76 (0.72–0.80)
Model 2	1.00 (Ref.)	0.95 (0.85–1.05)	0.81 (0.73–0.91)	0.73 (0.65–0.82)	1.536 × 10^−9^	0.84 (0.80–0.88)
Energy-adjusted fiber (g/d)	<14.23	≥14.23 & <17.43	≥17.43 & <20.98	≥20.98		
Cases/total person-years	905/541,220	573/545,731	556/546,696	514/545,932		2548/2,179,579
Model 1	1.00 (Ref.)	0.67 (0.60–0.75)	0.66 (0.59–0.73)	0.61 (0.54–0.68)	7.068 × 10^−20^	0.78 (0.74–0.82)
Model 2	1.00 (Ref.)	0.74 (0.66–0.82)	0.76 (0.68–0.84)	0.72 (0.64–0.80)	2.638 × 10^−9^	0.85 (0.81–0.89)

^a^ Energy adjustment for carbohydrates was conducted using the residual method. ^b^ Model 1: adjusted for age and sex; ^c^ Model 2: model 1 adjustments plus ethnicity, household income, education, BMI, smoking status, alcohol drinking status, and physical activity. Abbreviations: BMI, body mass index; IQR, interquartile range; Ref., Reference.

## Data Availability

Data will be made available on request.

## Supplementary Materials

The following supporting information can be downloaded at: https://www.mdpi.com/article/10.3390/nu16172883/s1. Figure S1: Flow diagram for participant inclusion; Figure S2: Proportion of blood or urine biomarkers mediating the association between dietary carbohydrates and gout; Table S1: Description of types and sources of dietary carbohydrates; Table S2: Information of SNPs used for gout genetic risk score construction; Table S3: Detailed information for biomarkers used in the present study; Table S4: Correlation matrix of different dietary carbohydrates; Table S5: Sensitivity analyses excluding participants who were diagnosed with gout within the first two year of follow-up; Table S6: Sensitivity analyses excluding participants diagnosed with gout before the last 24-h dietary assessment; Table S7: Sensitivity analyses restricting participants with at least two 24-h dietary assessments; Table S8: Sensitivity analyses restricting participants with fairly typical diet during 24-h dietary assessments; Table S9: Sensitivity analyses excluding participants with extreme energy intake; Table S10: Sensitivity analyses additionally adjusted for prevalent diabetes, prevalent cardiovascular disease, and prevalent hypertension; Table S11: Sensitivity analyses additionally adjusted for medication use; Table S12: Sensitivity analyses of the associations between dietary carbohydrates and the risk of gout after multiple imputations for missing covariates; Table S13: Stratified analyses of the associations between dietary carbohydrates and the risk of gout by age; Table S14: Stratified analyses of the associations between dietary carbohydrates and the risk of gout by sex; Table S15: Stratified analyses of the associations between dietary carbohydrates and the risk of gout by body mass index; Table S16: Association between genetic risk score and the risk of gout; Table S17: Additive and multiplicative interaction effects between dietary carbohydrates and genetic risk on the risk of gout; Table S18: Associations between energy-adjusted dietary carbohydrates and blood or urine biomarkers; Table S19: Associations between blood or urine biomarkers and the risk of gout; Table S20: Mediation effects of blood or urine biomarkers in the associations between energy-adjusted carbohydrates intakes and gout.
